# Towards a pituitary apoplexy classification based on clinical presentation and patient journey

**DOI:** 10.1007/s12020-022-02983-3

**Published:** 2022-01-24

**Authors:** M. C. Guijt, A. H. Zamanipoor Najafabadi, I. C. Notting, A. M. Pereira, M. J. T. Verstegen, N. R. Biermasz, W. R. van Furth, K. M. J. A. Claessen

**Affiliations:** 1 Dept. of Medicine, Division of Endocrinology, and Centre for Endocrine Tumors Leiden, Leiden University Medical Center, Leiden, the Netherlands; 2 Leiden University Medical Center, Haaglanden Medical Center and Haga Teaching Hospitals, University Neurosurgical Center Holland, Leiden and The Hague, the Netherlands; 3 Dept. of Ophthalmology, Leiden University Medical Center, Leiden, the Netherlands

**Keywords:** Pituitary apoplexy, Classification system, Spectrum of disease, Acute, Sub-acute and non-acute

## Abstract

**Purpose:**

The condition of pituitary apoplexia contains the clinical spectre from life-threatening emergency to asymptomatic self-limiting course, which partly determines diagnostic delay and management. Outcome evaluation of course and management of pituitary apoplexia is hampered by the diverse presentation of this condition and requires appraisal. This study aimed to describe the patient journey, clinical presentation, and management of various types of pituitary apoplexy in a new classification to facilitate future outcome evaluation and identify unmet needs in the care process.

**Methods:**

A single-center retrospective patient chart study was conducted between 2005–2021 (*N* = 98). Outcome measures were clinical symptoms at first presentation in hospital, being headache, consciousness, visual acuity, visual field defects (VFD), ophthalmoplegia, nausea, vomiting, fever, and hypopituitarism and care process characteristics.

**Results:**

Mean age was 47.6 ± 16.6 years (51.0% male). We describe their patient journey and identified three different types, differing in clinical presentation, in-hospital route, and final treatment, e.g., Acute (type A, 52%), Subacute (type B, 22.5%), and Non-acute (type C, 25.5%). Type A generally presents with acute onset headaches, VFD, or ophthalmoplegia emergency setting, with lowest mean visual acuity of both eyes and frequent hypocortisolism.

**Conclusions:**

Pituitary apoplexy can be approached as a spectrum of disease with 3 main subtypes, with a different initial presentation, different in-hospital route resulting in different management. Acknowledging subtypes with particular needs for (emergency) referrals to Pituitary Tumors Center of Excellence (PTCOE) will serve patient care improvements, outcome evaluations and address areas for improvement.

## Introduction

Pituitary apoplexy is a rare condition, defined as a hemorrhage or infarction of a pituitary adenoma. Patients typically present with acute or subacute symptoms of headache, decreased vision, visual field defects (VFD), or ophthalmoplegia, sometimes combined with decreased consciousness or fever without obvious infectious origin, and failure of one or more pituitary hormone axes [1, 2]. Patients presenting with these classical symptoms of pituitary apoplexy often require emergency intervention to prevent further progression and when possible, to improve neurological and endocrinological deficits [3].

It is increasingly acknowledged that not all patients present with the above-described well-known presentation of acute symptoms. Recently, Iqbal et al. described pituitary hemorrhage and infarction as a spectrum of disease, including a subacute variant of pituitary apoplexy [4]. Pituitary apoplexy e.g., signs of hemorrhage or infarction may be first detected by neuroradiologists as an incidental finding, so a proportion may stay undiagnosed and/or present with symptoms that mimic a non-apoplectic pituitary mass. In apoplexy patients with less-acute headache and visual complaints, e.g., not seen at the emergency ward, it is difficult to make the correct and timely diagnosis, given the broad differential diagnosis for headache symptoms. In addition, management is different from those with the emergency presentation, and therefore they have different needs in the care trajectory and should be discriminated from acute apoplexy in outcome evaluations.

Since all subtypes of apoplexy are ultrarare and with the variety of symptoms at presentation, healthcare workers of a broad range of disciplines are involved in the pre-diagnostic trajectory and are key players for prompt referral to the expert team. If no dedicated pituitary MRI is performed, the diagnosis pituitary apoplexy can be easily missed on general imaging for acute headache [5]. Many patients with apoplexy have been sent home initially with a misdiagnosis, after exclusion of a subarachnoid hemorrhage (SAB) or cerebral venous sinus thrombosis (CVST), since the computed tomography scan (CT-scan) did not reveal the pituitary apoplexy. Consequently there can be a referral delay to a center with specialized pituitary care with significant consequences for ultimate endocrine and ophthalmological outcome.

It is important to establish a classification method for pituitary apoplexy that acknowledge subtypes for outcome evaluation and improvement of the care path with attention to the path prior to arrival in a PTCOE for early recognition and prevention of unnecessary treatment delays in these patients. Nowadays, there are very limited classification methods for pituitary apoplexy [6, 7]. The UK guideline for pituitary apoplexy describes patients with a non-classical symptom presentation as “subclinical pituitary apoplexy” [7], but patients series of outcomes generally take all apoplexies together.

We aim to report our consecutive cases of apoplexy in the context of clinically identified subtype with a focus on presentation and care trajectory, distinguishing between acute, subacute, and non-acute pituitary apoplexy presentation. We evaluate the accompanying care process and the patient journey of the different subtypes of patients to identify possibilities to improve the care of these different patient groups that have own challenges, including timely referral to a specialized center.

## Methods

### Study design and patient selection

#### Study design

This study was conducted as a single-center retrospective patient chart study. Data on the clinical symptoms at hospital entry and data on the organization of the care process were obtained from electronic patient files at Leiden University Medical Centre (LUMC), which is a tertiary referral center for pituitary care with a dedicated pituitary care path, an expertise center within the European Reference Network on Rare Endocrine Conditions (Endo-ERN) [8]. Patients were selected from existing local patient registries and crosschecked via the LUMC radiological information system. Approval for this study was granted by the institutional review board of LUMC.

#### Patient selection

Between April 1 and May 31, 2021, a total of 780 possible apoplexy patients were screened: 709 of them were selected from a LUMC database including all operated patients for a pituitary adenoma, whereas 71 additional patients were identified by a search for pituitary apoplexy in the conclusion texts of radiology reports in the LUMC patient information system between 2005 and 2021. Inclusion criteria for the present study were [1]: clinical symptoms at initial presentation [2], MRI or CT-scan of the pituitary suggestive of pituitary apoplexy [3], sufficient clinical data available in the LUMC patient record, and [4] sufficient clinical data on the care process prior to referral to the LUMC. Based on the first two criteria, we included a total of 103 patients. Since 5 patients could not meet criteria 3 and 4, they were excluded from this study, bringing the total number of included patients in this study to 98.

#### Classification of pituitary apoplexy

For this study, we proposed and used the following classification system for pituitary apoplexy based on the initial patients presentation with (A) acute, (B) subacute, and (C) non-acute symptoms. We based this classification system on clinical experience with apoplexy patients in our specialized center and the recently described subacute apoplexy presentation by Iqbal et al. [4]. Generally, patients present at different caregivers based on the acuteness of symptoms. In the organization of the Dutch health care system, the general practitioner plays a major role, because he/she determines whether and within what time frame the patient is referred to specialist care in the hospital, and in emergency situations the ER is accessible directly and in those cases patients are usually first seen by ER physician or neurologist. Patients were in the following three newly defined categories of pituitary apoplexy (Table 1).

**Table 1:** Definition of the acute, subacute, and non-acute pituitary apoplexy subtype according to the ABC classification system

Subtype	Clinical definition
Type A: Acute pituitary apoplexy	Patients always have a clear sudden onset of pituitary apoplexy related symptoms (e.g., acute onset of severe headache, VFD, decrease in VA, and/or ophthalmoplegia) within a few hours to 3 days that require immediate ER assessment
Type B: Subacute pituitary apoplexy	Patients have an acute onset of pituitary apoplexy related symptoms (e.g., both acute and less acute onset of mild-severe headaches, decrease in VA, VFD, and/or ophthalmoplegia), and progression of these symptoms within a time period of 3 days to 2 weeks that require quick referral to an ER or outpatient PTCOE clinic
Type C: Non-acute pituitary apoplexy	Patients have experienced apoplexy related symptoms (e.g., non-acute onset mild-severe headaches, mild decrease in VA, VFD, and/or ophthalmoplegia) for weeks (at least longer than 2 weeks), with no obvious sudden moment of symptom onset that require immediate ER assessment

*VFD* visual field defects, *VA* visual acuity, *PTCOE* Pituitary Tumor Centers of Excellence

### Study parameters

#### Clinical parameters

The following clinical measures were evaluated, and compared between apoplexy type A, B, and C patients:

1. Clinical symptoms at first presentation in the hospital: headache (acute onset versus chronic), level of consciousness, mean visual acuity of both eyes (ODS), visual acuity of worst eye, VFD, CN. III, IV or VI palsy, nausea, vomiting, fever, hypopituitarism of at least one axis, hypocortisolism, hypothyroidism, hypogonadism, hyposomatotropism, hyperprolactinemia, and diabetes insipidus;
2. Time between onset symptoms and first presentation in hospital, time between first presentation and diagnosis, and time between first presentation and start treatment.

Visual acuity was measured using a Snellen chart by experienced optometrists and ophthalmologists [9]. VFD were identified by a Humphrey visual field analyser [10, 11]. For objectifying VFD, a cut-off value of 2 decibel below age-related normal was adopted by well-trained personnel [12]. Hypopituitarism was defined as clinically significant hormone deficiencies of at least one pituitary axis. Deficits of individual hormone axes were tested in accordance with the latest guidelines on testing for pituitary insufficiency [13, 14]. Time duration between different care process steps was calculated based on the calendar data that were noted in patient charts and referral letters from referring specialists. The time of starting treatment was defined as the day of surgery or the day of starting hydrocortisone supplementation for surgical and conservative treatment, respectively. Surgery was performed using an endonasal endoscopic approach [15]. Emergency and more elective surgery was defined as surgery within or after 3 days of arrival at the expertise center. The degree of consciousness was estimated from the report of the neurological examination at initial presentation to the hospital and was based on the Glasgow coma scale (GCS). Normal consciousness was defined as a GCS of 15; slightly lowered consciousness as a GCS of 13–14; lowered consciousness as a GCS of 8–12; and coma as a GCS below 8 [16].

#### Process measures

The following measures regarding the journey and management were evaluated and compared between apoplexy type A, B, and C patients: (1) consulting specialism at first presentation in hospital; (2) working diagnosis at first presentation; (3) location of first presentation inside the hospital; (4) type of hospital (referral hospital or pituitary centre); (5) hospital administration and emergency decompression after first presentation; (6) time between first presentation in regional hospital and referral to an academic hospital with specialized pituitary care, (7) previous management (if any) and actual management.

**Fig. 1:**
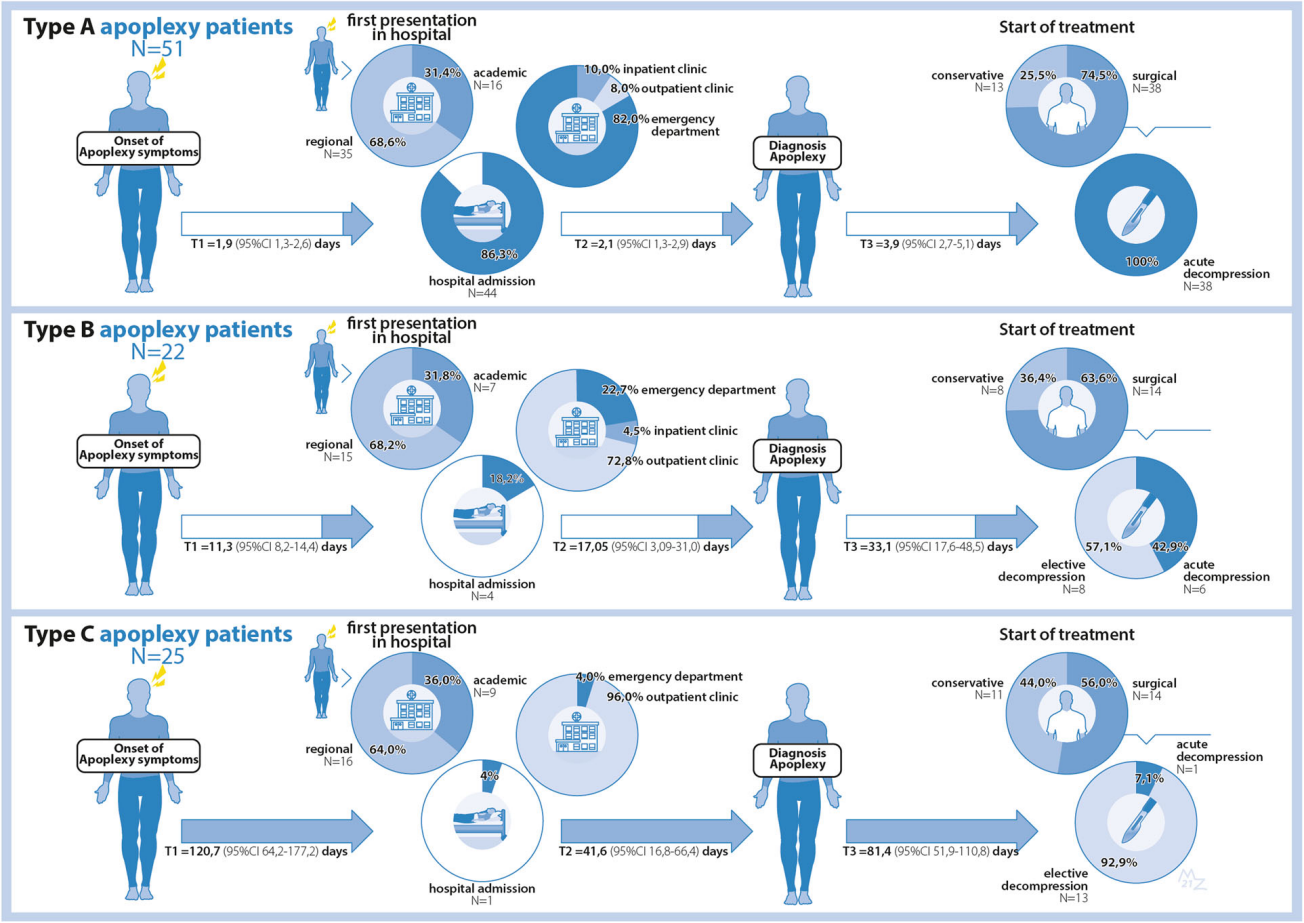
Patient journey throughout care process of apoplexy type A, B, and C patients. This figure illustrates the patient journey of apoplexy type A, B, and C patients throughout their care process until they reach treatment start. T1, time period in days between onset of apoplexy symptoms and first presentation in hospital (one way ANOVA between groups yielded *p* < 0.001); T2, time period in days between first presentation in hospital and moment of diagnosing pituitary apoplexy (one way ANOVA between groups yielded *p* < 0.001); T3, time period in days between first presentation in hospital and treatment start (one way ANOVA between groups yielded *p* < 0.001); Proportion of patients that were seen in a regional versus academic hospital at first presentation did not differ significantly between groups (chi-square yielded *p* = 0.917). Proportion of patients that were admitted to an inpatient ward in the hospital after first presentation did differ significantly between groups (chi-square yielded *p* < 0.001). The in hospital locations where patients were seen at their first hospital presentation significantly differed between the groups, with type A patients presenting primarily at the emergency department and almost all type C patients at the outpatient clinic (chi-square yielded *p* < 0.001). Proportion of patients that were treated either surgically or conservatively did not differ significantly between groups (chi-square yielded *p* = 0.248). Proportion of surgically treated patients that either had emergency (<3 days) or more elective (>3 days) surgery did differ significantly between groups (chi-square yielded *p* < 0.001)

Data on the care process organization of pituitary apoplexy patients were carefully extracted from patient charts and referral letters in the LUMC, and presented as a patient journey flowchart in the results section (Fig. 1).

### Statistical analysis

For statistical analyses of the collected data, SPSS (version 25.0 [IBM Corp., Armonk, New York, USA]) was used. Descriptive data were described as numbers (with percentages) and a mean or median, along with the standard deviation (SD) and interquartile range (IQR), respectively. An one-way ANOVA analysis or chi-square test was used to compare numeric or categorical data of three different groups. *p* < 0.05 was considered statistically significant.

## Results

### Baseline characteristics

A total of 98 patients were included (mean age 47.6 ± 16.6 years, 51.0% male). Fifty-one (52.0%) patients were classified as an acute pituitary apoplexy type A, 22 (22.5%) patients a subacute type B and 25 (25.5%) patients a non-acute type C (Table 2). Remarkably, the apoplexy type A group had a higher proportion of men compared to the other subtypes (type A 64.7%; type B 36.4%; type C 36.0% (*p* = 0.019)). Most patients had a non-functioning adenoma, irrespectively of apoplexy type, followed by prolactinoma. Most patients (67.3%) had not yet a known pituitary tumor diagnosed at onset of apoplexy complaints. From the patients with a known adenoma, 37.6% received treatment prior to apoplexy symptom onset (31.3% pharmacological treatment, 6.3% surgical treatment). Of patients with a known adenoma the classification was as follows: type A (53.1%), type B (25.0%) and type C (21.9%).

**Table 2:** Baseline characteristics of type A, B and C pituitary apoplexy patients

Characteristics at baseline	Type A *N* = 51	Type B *N* = 22	Type C *N* = 25	Total *N* = 98
Age at diagnosis, years (SD)	50.0 (16.9)	46.4 (13.0)	43.6 (18.6)	47.6 (16.6)
Male (N)	64.7 (33/51)	36.4 (8/22)	36.0 (9/25)	51.0 (48/98)
Mean BMI, kg/m² (SD)	27.8 (4.83)	27.6 (5.68)	30.2 (8.42)	28.4 (6.20)
Hypertension (*N*)	27.5 (37/51)	31.8 (7/22)	12.0 (3/25)	24.5 (24/98)
Diabetes mellitus (*N*)	15.7 (8/51)	0.0 (0/22)	8.0 (2/25)	10.2 (10/98)
Smoking (*N*)	12.0 (6/50)	13.6 (3/22)	12.0 (3/25)	12.4 (12/97)
Any ophthalmic comorbidity (*N*)	13.7 (7/51)	13.6 (3/22)	12.0 (3/25)	13.3 (13/98)
Anticoagulant use (*N*)	13.7 (7/51)	13.6 (3/22)	20.0 (5/25)	15.3 (15/98)
GnRH agonist use (*N*)	5.9 (3/51)	0.0 (0/22)	4.0 (1/25)	4.1 (4/98)
Dopamine agonist use (*N*)	7.8 (4/51)	13.6 (3/22)	16.0 (4/25)	11.2 (11/98)
Surgery* or trauma <5 days onset symptoms	9.8 (5/51)	0.0 (0/22)	0.0 (0/25)	5.1 (5/98)
Pregnancy	7.8 (4/51)	4.5 (1/22)	8.0 (2/25)	7.1 (7/98)
Known adenoma prior to apoplexy (*N*)	33.3 (17/51)	36.4 (8/22)	28.0 (7/25)	32.7 (32/98)
Micro adenoma (*N*)	6.0 (3/50)	4.5 (1/22)	17.4 (4/23)	8.4 (8/95)
Macro adenoma (*N*)	92.0 (46/50)	90.9 (20/22)	73.9 (17/23)	87.4 (83/95)
Giant adenoma (*N*)	2.0 (1/50)	4.5 (1/22)	8.7 (2/23)	4.2 (4/95)
NFA (*N*)	78.4 (40/51)	59.1 (13/22)	36.0 (9/25)	63.3 (62/98)
Prolactinoma (*N*)	11.8 (6/51)	18.2 (4/22)	36.0 (9/25)	19.4 (19/98)
RCC (*N*)	2.0 (1/51)	13.6 (3/22)	12.0 (3/25)	7.1 (7/98)
Cushing (*N*)	3.9 (2/51)	0.0 (0/22)	4.0 (1/25)	3.1 (3/98)
Acromegaly (*N*)	2.0 (1/51)	4.5 (1/22)	0.0 (0/25)	3.1 (3/98)
Sheehan (*N*)	2.0 (1/51)	0.0 (0/22)	4.0 (1/25)	2.0 (2/98)
Other (*N*)	0.0 (0/51)	4.5 (1/22)	8.0 (2/25)	3.1 (3/98)

Data are presented as mean ± SD or % (N) unless stated otherwise. *SD* standard deviation, *N* number, *GnRH* Gonadotropin-releasing hormone, *BMI* body mass index, *NFA* non-functioning adenoma, *RCC* Rathke’s cleft cyst

*two patients had major cardiothoracic surgery, one had major abdominal surgery

We were able to identify a potential triggering factor of pituitary apoplexy in 43 patients (42.9%), of whom, 15 patients (15.3%) used therapeutic anticoagulant treatment, 4 (4.1%) patients used a GnRH agonist for the treatment of prostate cancer and 11 patients (11.2%) used a dopamine agonist during onset of apoplexy symptoms. Furthermore, 5 patients (5.1%) had major surgery or head trauma within 5 days prior to symptom onset and another 7 patients (7.1%) were pregnant at the onset of apoplexy symptoms, although at different times of the gestational age. In the other 55 patients, no triggering factors could be identified. The distribution of potential eliciting factors among the different apoplexy subtypes is shown in Table 2.

### Clinical symptoms at first hospital presentation of pituitary apoplexy subtypes

An overview of initial clinical symptoms of the different apoplexy subtypes is depicted in Table 3. In summary, upon entering the hospital (in the vast majority at the emergency ward) patients from group A suffered more often from acute onset headache and vomiting, and there is a shift in the proportion of patients who reported having acute onset headaches to non-acute onset headaches between apoplexy subtypes (type A: 98.0% acute *vs* 2.0% non-acute; type B: 55.0% acute *vs* 45.0% non-acute; type C: 10.5% acute *vs* 89.5% non-acute (*p* < 0.001)). All type B and C patients had normal consciousness at hospital entry, whereas 6 (12.0) and 3 (6.0%) patients with apoplexy type A had slightly lowered (GCS 13–14) and lowered consciousness (GCS 8–12), respectively. Type A patients presented most frequently with N. III, IV or VI failure compared to type B or type C (type A 66.7%; type B 27.3%; type C 8.0% (*p* < 0.001)).

**Table 3:** Symptoms at first presentation in hospital of pituitary apoplexy type A, B, and C patients

Symptom at first hospital presentation	Type A *N* = 51	Type B *N* = 22	Type C *N* = 25	Total *N* = 98	*p*-value
Any headache (*N*)	98.0 (49/50)	90.9 (20/22)	76.0 (19/25)	90.7 (88/97)	0.008
Acute onset < 3 days (*N*)	98.0 (48/49)	55.0 (11/20)	10.5 (2/19)	69.3 (61/88)	<0.001
No acute onset (*N*)	2.0 (1/49)	45.0 (9/20)	89.5 (17/19)	30.7 (27/88)	<0.001
Normal consciousness (*N*)	82.0 (41/50)	100 (22/22)	100 (25/25)	90.7 (88/97)	0.053
Slightly lowered (*N*)	12.0 (6/50)	0.0 (0/22)	0.0 (0/25)	6.2 (6/97)	0.053
Lowered (*N*)	6.0 (3/50)	0.0 (0/22)	0.0 (0/25)	3.1 (3/97)	0.053
Visual acuity ODS (SD)	0.72 (0,39)	0.90 (0.32)	1.02 (0.12)	0.84 (0.35)	0.002
Visual acuity of the worst eye (SD)	0.62 (0,44)	0.82 (0.36)	0.96 (0.16)	0.76 (0.39)	0.002
VFD (*N*)	66.7 (34/51)	63.6 (14/22)	32.0 (8/25)	57.1 (56/98)	0.013
CN. III, IV or VI palsy (*N*)	66.7 (34/51)	27.3 (6/22)	8.0 (2/25)	42.9 (42/98)	<0.001
Nausea (*N*)	58.8 (30/51)	40.9 (9/22)	8.0 (2/25)	41.8 (41/98)	<0.001
Vomiting (*N*)	56.9 (29/51)	27.3 (6/22)	8.0 (2/25)	37.8 (37/98)	<0.001
Fever without focus (*N*)	24.0 (12/50)	4.5 (1/22)	8.0 (2/25)	15.5 (15/97)	0.053
Loss of at least one axis (*N*)	80.4 (41/51)	68.2 (15/22)	60.0 (15/25)	72.4 (71/98)	0.153
Hypocortisolism (*N*)	62.7 (32/51)	31.8 (7/22)	36.0 (9/25)	49.0 (48/98)	0.017
Hypothyroidism (*N*)	64.7 (33/51)	59.1 (13/22)	40.0 (10/25)	57.1 (56/98)	0.121
Hypogonadism (*N*)	56.9 (29/51)	45.5 (10/22)	44.0 (11/25)	51.0 (50/98)	0.481
Hyposomatotropism (*N*)	39.2 (20/51)	18.2 (4/22)	15.0 (4/25)	28.6 (28/98)	0.052
Hyperprolactinemia (*N*)	11.8 (6/51)	36.4 (8/22)	44.0 (11/25)	25.5 (25/98)	0.004
Diabetes insipidus (*N*)	5.9 (3/51)	4.5 (1/22)	4.0 (1/25)	5.1 (5/98)	0.932

Data are presented as mean ± SD or % (*N*) unless stated otherwise. *SD* standard deviation, *N* number, *ODS* both left and right eye, *VFD* Visual field defects, C*N* Cranial nerve

Mean visual acuity ODS was compressed at initial presentation in apoplexy type A and B patients, but not in type C patients (mean visual acuity ODS ± SD: type A 0.72 ± 0.39; type B 0.90 ± 0.32; type C 1.02 ± 0.12 (*p* = 0.002)). VFD occurred more frequently in type A and B patients than in type C patients (type A 66.7%; type B 63.6%; type C 32.0% (*p* = 0.013)).

At initial hospital presentation, 71 patients (72.4%) had failure of at least one pituitary axis. Patients with the acute apoplexy type A were much more likely to have failure of the cortisol axis than patients with the subacute or non-acute apoplexy type (type A 62.7%; type B 31.8%; type C 36.0% (*p* = 0.017)), whereas the non-acute apoplexy patients had elevated prolactin more often at first presentation (type A 11.8%; type B 36.4%; type C 44.0% (*p* = 0.004)). No significant differences were seen in the other pituitary axes.

### Patient journey and care process organization of pituitary apoplexy subtypes

Figure 1 shows the patient journey of type A, B, and C patients throughout their care process from onset of initial complaints to start of initial treatment. The time period between onset of symptoms (as noted in records) and first hospital presentation differed significantly between the three groups (type A 1.9 (95%CI 1.3–2.6) days); type B 11.3 (95%CI 8.2–14.4) days; type C 120.7 (95%CI 64.2–177.2) days (*p* < 0.001). In case the first presentation was in a referral hospital, it took on average 4.4 days for type A patients (95%CI 1.2–7.6) before they were seen in our expertise center for pituitary care. For type B and C, this took 14.1 days (95%CI 5.3–22.8) and 53.8 days (95%CI 33.5–7.6), respectively (*p* < 0.001). Similarly the time between initial hospital presentation and diagnosis was greatest in type C patients, as was the time between first hospital presentation and start of initial treatment. The proportion of patients that underwent either conservative or surgical treatment, and, within the surgical group, had either emergency or elective surgery differed between the three patient groups. Of the type A patients, 25.5% were treated conservatively and 74.5% surgically, all of whom had an emergency procedure. Of the type B patients, 36.4% were treated conservatively and 63.6% surgically, of whom 42.9 and 57.1% underwent elective emergency surgery and elective surgery, respectively. In type C patients, 44.0% had conservative treatment and 56.0% surgery, and within the surgical group, 7.1% had emergency surgery, while 92.9% rather had more elective surgery.

**Fig. 2:**
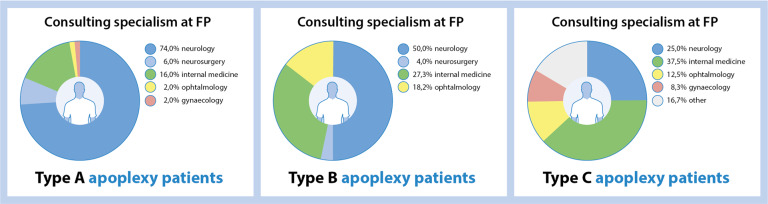
Consulting specialism at first hospital entry for apoplexy type A, B, and C patients. The consulting specialism at first presentation in the hospital significantly shifts between the different apoplexy types, from neurology is by far the largest proportion of type A patients to internal medicine and ophthalmology in type C patients (chi-square yielded *p* < 0.001). FP first presentation in hospital

With respect to the in-hospital presentation, Type A patients were usually seen at the emergency department by a neurologist (*see* Figs. 1 and 2), whereas type B patients were most often seen first at the neurology outpatient clinic. Type C patients were most often initially referred by their general practitioner to the outpatient clinic of the internal medicine and only a small minority of 4.0% was evaluated at the emergency department.

**Fig. 3:**
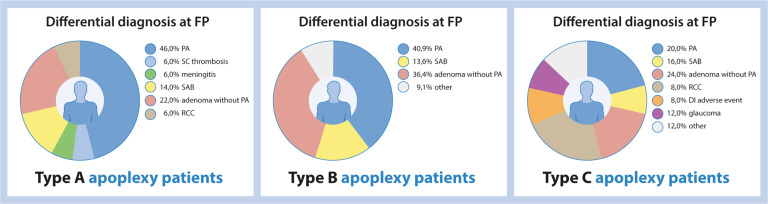
Differential diagnosis at first hospital entry for apoplexy type A, B, and C patients. The variation of differential diagnoses at first presentation in the hospital significantly differed between the groups (chi-square yielded *p* = 0.014). PA pituitary apoplexy; SC Thrombosis, sinus cavernous thrombosis; SAB subarachnoid hemorrhage; RCC Rathke’s cleft cyst

With respect to initial working diagnosis (Fig. 3), the combination of symptoms at hospital entry was recognized as a pituitary apoplexy in 46.0% of type A patients, whereas in type B and C patients, the clinical presentation was recognized as pituitary apoplexy in, respectively, 40.9 and 20.0% of cases.

## Discussion

Our study stresses the existence of subtypes of pituitary apoplexy, which we believe are important to discriminate for outcome evaluation and management. It may create more awareness for the preferred referral trajectory in emergencies and facilitate the identification of patients with pituitary apoplexy, who present with a less typical combination of symptoms. Moreover, acknowledgement of the spectrum of symptoms with which apoplexy patients can present, using the ABC subtypes, facilitates timely and proper treatment of apoplexy patients. Based on insight of presentation, e.g., type A via emergency ward seen by neurologist, type B, seen by neurologist or endocrinologist via the outpatient clinic, and type C via the regular route of pituitary adenoma care, we can further develop the prediagnostic trajectory. With respect to treatment, it is notable that type A patients had emergency surgery more often, while type B and C increasingly received conservative treatment or more elective surgery. Despite this, there is considerably heterogeneity within subgroups which deserves future attention of prospective studies including validation of the different apoplexy subtypes and outcome evaluation of conservative management versus emergency surgery as well as emergency surgery versus elective surgery.

Several studies have already attempted to compare the outcomes of conservative and surgical treatment of pituitary apoplexy (and its timing) [17–19]. The heterogeneity of apoplexy patients, however, prevented a proper comparison between these treatment options in the past. Introducing this classification method paves the way to better evaluate outcomes of treatment within the apoplexy subtypes and thereby personalize treatment.

By classifying apoplexy patients according to the time of onset and severity of symptoms, more justice is done to the new spectrum of disease approach to pituitary apoplexy as recently proposed by Iqbal et al. [4]. Iqbal et al. described the underestimation of the subacute apoplexy subtype. In addition to that, our study showed that besides a subacute presentation of pituitary apoplexy as described by Iqbal et al, there is also a substantial group of patients who do not present acutely or subacutely at all. Since management in these cases will be very different from acute apoplexy it is important that subtypes are not mixed in outcome evaluations. Moreover, our study is first to detail the different prediagnostic care paths and treatment of subtypes. Future prospective studies are needed to validate this apoplexy classification system.

Remarkably, in type A patients, these onset of headaches was acute in 98.0% of patients, while in type C patients the onset was not acute, and in type B patients the headache onset was half acute and half non-acute. Since type C apoplexy patients had no acute onset of headache, it is difficult to distinguish these patients in particular from patients with a pituitary adenoma without apoplexy. It is possible that this difficult distinction has led to a long-standing underestimation of this non-acute type of pituitary apoplexy. We recommend to actively consider the diagnosis of pituitary apoplexy in all patients with a pituitary adenoma and headache complaints, also when patients do not have the acute onset and severe headaches, which are seen in type A patients. Further outcome research is needed whether these non-acute cases harbor a different course than those with a pituitary adenoma without apoplexy. It is of note that a high proportion of non-acute cases was operated in an elective setting.

Regarding initial endocrinological status, type A patients had more frequent failure of the cortisol axis. For the other axes, there was no significant difference in the proportion of patients with failure between the different subtypes. This would recommend low threshold preventive hydrocortisone replacement as patients will experience stress. Zayour et al. described an inverse relationship between prolactin levels and the likelihood of postoperative recovery of pituitary function after surgery [20], since preoperative prolactin levels are related to the degree of necrosis-induced damage of pituitary cells. In our study, we observed that type A patients were significantly less likely to have hyperprolactinemia at first presentation than type B or C patients. For future research, it would be interesting to investigate whether prolactin values are predictive of postoperative recovery of the pituitary axes in the different subtypes of apoplexy patients.

The prevalence of persistent hypopituitarism is considered quite high in comparison with non-apoplectic pituitary adenomas. Therefore, future research on the best treatment of pituitary apoplexy subtypes should also focus on achieving the best possible endocrinological outcome, comparing conservative treatment, emergency surgery, and elective surgery within the subtypes.

It is noteworthy that type A patients were more often male than type B or C patients. Literature shows that the male sex is a risk factor for pituitary apoplexy [14]. Our results showed that this can be further specified: male gender was a risk factor for acute apoplexy, but not so much for apoplexy in general. The exact etiology of pituitary apoplexy is unknown. According to an overview article of Albani et al., known eliciting factors for pituitary apoplexy include the use of anticoagulants, dopamine agonists, estrogen supplementation, GnRH agonists, pregnancy, recent traumatic brain injury, or major orthopedic and cardiac surgery [21]. Although our study design is not suitable to prove causality, we found potential triggering factors for pituitary apoplexy in 42 patients (42.9%), mainly the use of therapeutic anticoagulation, dopamine agonists, pregnancy, and recent major surgery or head trauma. Interestingly, all patients with recent major surgery or head trauma belonged to apoplexy subtype A. Future studies should aim to better identify triggering factors specific to the different subtypes of pituitary apoplexy.

In addition to the differences in symptom presentation at hospital entry, our study also showed for the first time that patients of the different apoplexy subtypes go through a different type of care process with different initial working diagnosis, and eventually different treatments. Due to the differences in care pathways before diagnosis, patients with different subtypes of pituitary apoplexy arrived at our specialized center in a different way. Our data emphasize that many physicians do not primarily consider the diagnosis of pituitary apoplexy in case of a subacute or non-acute symptom presentation (types B and C). Since many patients with more chronic complaints may never be recognized as having pituitary apoplexy, this may not lead only to underreporting and undertreatment of pituitary apoplexy, but also to persistent symptoms and shortage of specialized treatment.

**Fig. 4:**
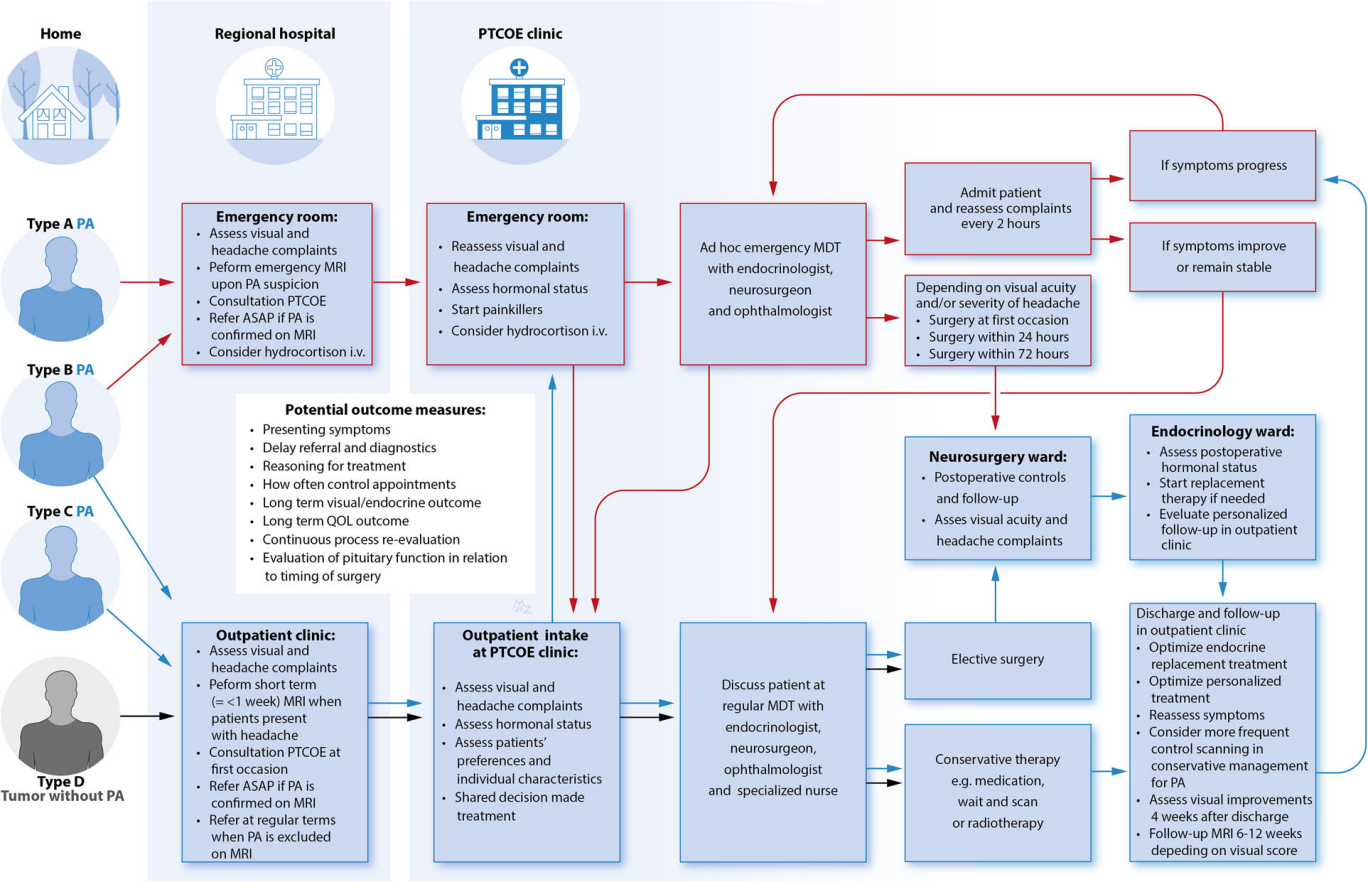
Proposal for care process improvements for pituitary apoplexy. This figure addresses a proposal for care process improvement with regard to pituitary apoplexy. The suggested possible care paths (red and blue) for each subtype are demonstrated in comparison to the care path of patients with a pituitary tumor without PA (grey). PA pituitary apoplexy; PTCOE Pituitary Tumor Center of Excellence; MDT Multidisciplinary team meeting; ASAP As soon as possible; MRI Magnetic resonance imaging

Thus, the most important unmet needs in the pituitary apoplexy care process are: (1) delay in referral to a Pituitary Tumors Center of Excellence (PTCOE), (2) underdiagnosis of the B and C subtype, since physicians do not recognize subacute and non-acute complaints as pituitary apoplexy; (3) lack of long-term outcome evaluation of different treatment modalities within subtypes. Therefore, we suggest three different care paths for the subsequent types of pituitary apoplexy in order to improve quick diagnosis, to speed up referral process to a PTCOE, and, in time, to optimize personalized treatment for pituitary apoplexy (*see* Fig. 4). These care paths take into account that symptoms might decrease or progress over time, on which should be acted. Once patients arrive at the PTCOE, crosslinks between the different care paths can help to continuously evaluate the process and outcomes and impact decision making with respect to continuing conservative management or proceed to surgery. By doing so, in the future, an optimal individualized treatment can be achieved for pituitary apoplexy patients and different treatment options can be properly compared, which has been very hard to do in the treatment of pituitary apoplexy up to now.

The strengths of this study are the large population size with a very limited amount of missing data. It is important to address some limitations of this study. First of all, there might be some selection bias, since all our patients were seen in a tertiary hospital eventually. It is plausible that many type B and C patients were not referred to our hospital at all. However, this actually further supports the need for more recognition of these subtypes of pituitary apoplexy. Second, given the retrospective design, we did not include patient experiences about their patient journey which could be very helpful to identify bottlenecks in the current care process organization. Third, care process organization differs between countries and conclusions about this are therefore hard to extrapolate to other countries with different care systems. Nevertheless, this was the first study to explore the patient journey of pituitary apoplexy patients (in the Netherlands), showing many opportunities for improvement of the care process for pituitary apoplexy.

In conclusion, we introduced a suitable classification system for pituitary apoplexy, underlining that pituitary apoplexy needs to be approached as a spectrum of disease, in which different patient subgroups not only had a different initial presentation, but also different in-hospital route and final treatment. We recommend this classification system in clinical practice for its potential to better and faster recognize subacute and non-acute pituitary apoplexy patients and make a suggestion how this classification can be implemented in a suitable care path for each pituitary apoplexy subtype. This classification method should be validated in future prospective studies, and it is important to further study whether treatment options need to be individualized for the respective apoplexy subtypes.

## Data Availability

Available upon request.
